# Sleep and Arousal Hubs and Ferromagnetic Ultrafine Particulate Matter and Nanoparticle Motion Under Electromagnetic Fields: Neurodegeneration, Sleep Disorders, Orexinergic Neurons, and Air Pollution in Young Urbanites

**DOI:** 10.3390/toxics13040284

**Published:** 2025-04-08

**Authors:** Lilian Calderón-Garcidueñas, Fredy Rubén Cejudo-Ruiz, Elijah W. Stommel, Angélica González-Maciel, Rafael Reynoso-Robles, Héctor G. Silva-Pereyra, Beatriz E. Pérez-Guille, Rosa Eugenia Soriano-Rosales, Ricardo Torres-Jardón

**Affiliations:** 1 Biomedical Sciences, The University of Montana, Missoula, MT 59812, USA; 2 Escuela de Enfermeria, Universidad Autónoma de Piedras Negras, Piedras Negras 26000, Mexico; 3 Instituto de Geofísica, Universidad Nacional Autónoma de México, Mexico City 04510, Mexico; ruben@igeofisica.unam.mx; 4 Department of Neurology, Geisel School of Medicine, Dartmouth-Hitchcock Medical Center, Lebanon, NH 03756, USA; elijah.w.stommel@hitchcock.org; 5 Instituto Nacional de Pediatría, Mexico City 04530, Mexico; agonzalezmaciel@yahoo.com (A.G.-M.); reynosoraf@yahoo.com (R.R.-R.); bettyepg@yahoo.com (B.E.P.-G.); resr62@yahoo.com.mx (R.E.S.-R.); 6 Department of Advance Materials, Instituto Potosino de Investigación Científica y Tecnológica AC, San Luis Potosi 78216, Mexico; hector.silva@ipicyt.edu.mx; 7 Instituto de Ciencias de la Atmósfera y Cambio Climático, Universidad Nacional Autónoma de México, Mexico City 04510, Mexico; rtorres@unam.mx

**Keywords:** anthropogenic ferrimagnetic nanoparticles, air pollution, Alzheimer’s, orexinergic neurons, sleep disorders, Parkinson’s, obesity, sleep/awake hubs, electromagnetic fields

## Abstract

Air pollution plays a key role in sleep disorders and neurodegeneration. Alzheimer’s disease (AD), Parkinson’s disease (PD), and/or transactive response DNA-binding protein TDP-43 neuropathology have been documented in children and young adult forensic autopsies in the metropolitan area of Mexico City (MMC), along with sleep disorders, cognitive deficits, and MRI brain atrophy in seemingly healthy young populations. Ultrafine particulate matter (UFPM) and industrial nanoparticles (NPs) reach urbanites’ brains through nasal/olfactory, lung, gastrointestinal tract, and placental barriers. We documented Fe UFPM/NPs in neurovascular units, as well as lateral hypothalamic nucleus orexinergic neurons, thalamus, medullary, pontine, and mesencephalic reticular formation, and in pinealocytes. We quantified ferromagnetic materials in sleep and arousal brain hubs and examined their motion behavior to low magnetic fields in MMC brain autopsy samples from nine children and 25 adults with AD, PD, and TDP-43 neuropathology. Saturated isothermal remanent magnetization curves at 50–300 mT were associated with UFPM/NP accumulation in sleep/awake hubs and their motion associated with 30–50 µT (DC magnetic fields) exposure. Brain samples exposed to anthropogenic PM pollution were found to be sensitive to low magnetic fields, with motion behaviors that were potentially linked to the early development and progression of fatal neurodegenerative diseases and sleep disorders. Single-domain magnetic UFPM/NPs in the orexin system, as well as arousal, sleep, and autonomic regions, are key to neurodegeneration, behavioral and cognitive impairment, and sleep disorders. We need to identify children at higher risk and monitor environmental UFPM and NP emissions and exposures to magnetic fields. Ubiquitous ferrimagnetic particles and low magnetic field exposures are a threat to global brain health.

## 1. Introduction

Exposure to particulate matter (PM) air pollution in the prenatal and postnatal stages has been independently associated with adverse cognitive, internalizing and externalizing behaviors, brain structural abnormalities, sleep disorders, and mental health outcomes in children, adolescents, and adults [1,2,3,4,5,6,7,8,9,10]. Fine particulate matter (PM_2.5_) with a diameter ≤ 2.5 μm is the toxicological proxy for PM, which includes the ultrafine PM ≤ 100 nm (UFPM) fraction. Our work has demonstrated that exposure to UFPM and industrial nanoparticles (NPs) is a key trigger for neurodegeneration in pediatric age groups [11,12,13,14,15,16].

The US EPA has a lower 2024 annual National Ambient Air Quality Standard for PM_2.5_ at 9.0 μg/m^3^ [17]. We have millions of *de facto* USA residents living in PM_2.5_ non-attainment regions, with UFPM/NPs that are capable of crossing all biological barriers and reaching the brain, starting *in utero*. Fossil fuel combustion- and friction-generated UFPM emissions are ubiquitous across the USA, and these are not uniformly or even directly regulated [18]. Individual radio-frequency electromagnetic field (RF-EMF) exposure has increased in the last few years, with mobile downlink, mobile uplink, broadcast, and 5G-New Radio contributing to the highest exposures; these have a detrimental impact on the brain and heart [19,20,21,22,23]. Researchers have serious health concerns about new technologies and their short- and long-term consequences [19,20,21,22,23].

The metropolitan area of Mexico City (MMC) is an example of an exposure chamber where 22 million people breathe PM_2.5_ concentrations that are above the USEPA annual standard from conception to death, and where brain structural changes (supra and infratentorial), brain atrophy, cognition and olfaction deficits, peripheral and central auditory system dysfunction, gait and balance disturbances, fall risks, high risk of suicide, and sleep disorders are all documented in seemingly clinically healthy children and young adults [24,25,26,27,28,29,30,31,32]. UFPM/NPs have been identified in placentas of all gestational ages and in brains from postconceptional weeks 12–15, strongly suggesting that brain damage could start *in utero* [16].

Researchers around the world have identified neurodegeneration in the pathophysiology of sleep disorders, including idiopathic REM sleep behavior disorder (iRBD), a parasomnia associated with Parkinson’s disease (PD), and Lewy Body dementia (LBD) [33,34,35,36,37,38]. Chemical pollutants, including air pollution, Gulf War exposures, endocrine disruptors, metals, pesticides, and solvents, have also been associated with sleep outcomes [39,40]. Sleep-disordered breathing has been identified in Boston, MA low-income children exposed to high indoor PM_2.5_ levels with odds 3.53-fold and the association persisting after adjustments for physical activity, outdoor PM_2.5_, environmental tobacco smoke, and health characteristics [40]. The brain areas involved in arousal, sleep, and autonomic and motor systems include cortical and subcortical regions [41,42,43,44,45,46,47,48]. The development of *in vivo* probabilistic MRI neuroimaging structural templates of key sleep and arousal hubs has enabled the localization of crucial nuclei in MRI images, which is key for future studies of sleep and arousal physiology and neuropathology [41,42,43,44,45,46,47,48].

Neuropathological hallmarks of AD, PD, and TDP-43 pathology, namely frontotemporal lobar degeneration (FTLD) and amyotrophic lateral sclerosis (ALS), have been identified in young MMC residents [11,12,13,14,15,32]. The neuropathology of these young urbanites has been linked to UFPM and industrial NPs [14,49]. Exogenous magnetic Fe-rich NPs resulting from fuel combustion and engineered titanium (Ti) nanorods from E-Waste, alongside Al, V, Ni, Hg, Co, Cu, Zn, Ag, Pt, Ce, La, Pr, and W, have been identified in fetal and term neural and vascular cells and subcellular structures, i.e., mitochondria, endoplasmic reticulum, Golgi, lysosomes, and neuromelanin [11,12,13,14,15,32,49]. UFPM and NPs are cardiotoxic and neurotoxic, and their size allows them to cross all biological barriers, giving rise to oxidative, endoplasmic reticulum, and mitochondrial oxidative stress, neuroinflammation, DNA damage, protein aggregation, and misfolding [50,51,52].

We have described sleep, cognitive, olfactory, and auditory disturbances, brain MRI structural changes, and high suicide risk in young MMC urbanites, in keeping with neurodegenerative hallmarks observed in forensic autopsies [11,24,25,26,27,28,30,31,32]. The significant frontal, parietal and temporal, caudate, and cerebellar gray and white matter atrophy observed in MMC children and young adults [24,25] strongly suggest that neurodegenerative processes start in childhood and are in a disease continuum for several decades before cognitive impairment and neurological symptoms are in place.

In light of the substantial global information on PM pollution, brain development, neurodegeneration, sleep disorders [1,2,3,4,5,6,7,8,9,10,33,34,35,36,37,38,39,40], and neuropathology [11,12,13,14,15,16], there is an urgent need to explore key links between early environmental exposures and preventable short/long-term effects on the central nervous system (CNS) [18]. This study examines three significant aspects of sleep, arousal, autonomic, and motor subcortical and cortical hubs in pediatric and young adult urban populations: (1) Magnetic brain UFPM/NPs measurements in megacity residents, (2) Identification of ferrimagnetic particles and their behavior under magnetic AC and DC fields in sleep and arousal regions, and (3) Ultrastructural pathology associated with brain UFPM/NPs and representative samples of energy-dispersive X-ray spectrometry (EDX) in targeted orexinergic neuron lateral hypothalamic nucleus, pinealocytes, thalamic, medullary, pontine, and mesencephalic reticular formation regions.

We hypothesized that selected brain hubs in young MMC residents would display single domain ferrimagnetic UFPM/NPs with motion behavior influenced by ubiquitous magnetic AC and DC fields capable of inducing major structural cell damage.

Our study indicates magnetic particles in sleep, arousal, autonomic, and motor brain hubs are abundant in young urban residents, sensitive to the application of low magnetic fields, and key risk factors for pediatric development of neurobehavioral disorders.

Magnetophoresis results in sleep, awake, autonomic, and motor hubs are bridging the gap between anthropogenic air pollution, ubiquitous low magnetic fields, neurodegeneration, and behavioral/sleep/awake/autonomic/hypothalamic/obesity disorders, starting in childhood. Brain single-domain magnetic NPs are important anthropogenic early-life risk factors for sleep and eating disorders and neurodegeneration.

## 2. Materials and Methods

### 2.1. Study Cities and Air Quality

Metropolitan Mexico City (MMC) has 22 million residents chronically exposed to high concentrations of PM_2.5_ and NPs for the last 3 decades [53]. Measurements of heavy metal concentrations have been performed in street dust in MMC [54]. Metal concentrations and environmental ferrimagnetic particles have been studied, and their magnetic properties characterized [55,56,57,58,59,60,61,62,63,64,65,66,67].

### 2.2. Study Design and Brain Samples

The focus of our study was MMC children and young adults seemingly healthy at the time of their death. We had access to 164 brain forensic samples, including samples from 9 children aged 12.8 ± 6.4 years and 25 adults aged 35.4 ± 21.9 years. Cortical and subcortical brain regions were included (Table 1). All subjects had complete neuropathology, anatomical pathology, and toxicology studies [11,12,13,14,15,16,29,32,49]. The forensic study was approved by the Forensic Institute in Mexico City [Permit # 20/64/2003].

#### 2.2.1. Magnetic Experiments

We examined the ARM and SIRM as in previous publications [49]. Briefly, ARM is greatly sensitive to small single-domain magnetic grains [61,62]. IRM was acquired without changes in temperature and is useful for ferrimagnetic mineral (i.e., magnetite is typically saturated at 300–500 mT) identification and concentration [63,64,65,66]. A pulse magnetizer IM-10 (ASC Scientific, Narragansett, RI, USA) allowed for up to 1000 mT direct fields and 300 mT (opposite direction) backward fields. SIRM was the IRM acquired at 1000 mT. Normalization to mass was performed for ARM and SIRM values. The S-ration was calculated as S_300_ = IRM_-300_/SIRM, using IRM 300 mT (IRM_300_) in a backward field.

#### 2.2.2. Transmission Electron Microscopy (TEM) and Energy-Dispersive X-Ray Spectrometry (EDX)

Three mm block brain samples—cut with ceramic knives and handled with plastic forceps, free from metal contamination—were used for TEM work. A Carl Zeiss Axioskop 2 PLUS microscope (Carl Zeiss, Dublin, CA, USA) equipped with an AxioVision REL 4.8 imaging system was the selected equipment. The focus of the brain evaluation documented the neurovascular unit, defined the location of electrodense UFPM/NPs, and described the structural changes in cell organelles. All samples were examined blind to case, and grids/tissue sections and grid areas were randomly selected and scanned. For the EDX, information regarding the chemical elements present in the samples was obtained through characteristic X-ray spectra [49].

#### 2.2.3. Light Microscopy and Orexin Immunohistochemistry

Forensic neuropathological diagnostic examination of the MMC 9 children and 25 adults is shown in Supplemental Table S1. The neuropathological findings have been reported previously along with the diagnostic criteria for AD, PD, FTLD, and ALS [11,12,13,14,15,29,32]. We used Anti-Orexin receptor 1/OX-1-R antibody-N terminal Abcam 224368 1:100 with a previous heat-mediated Ag retrieval citrate buffer pH 6 according to the Abcam (Waltham, MA, USA) protocol.

### 2.3. Statistical Analysis

We concentrated on a summary of the targeted magnetic variables.

## 3. Results

### 3.1. Air Pollution

Uncontrolled urban growth and environmental pollution have characterized MMC for the last three decades [53,67]. This study included MMC residents at 2200 m above sea level, in a 2000 km^2^ valley, exposed to daily toxic emissions from >60,000 industries and 6 million vehicles including heavy diesel vehicles not subjected to any regulatory standards. The main air pollutant is PM_2.5_, a heterogeneous mixture of multiple chemical components, including sulfate, nitrate, ammonium, organic carbon, elemental carbon, and trace elements from emission sources (i.e., heavy diesel vehicles and public transportation) and atmospheric processes. UFPM/NPs are key components of PM_2.5_ [17,53,54,55,56,57,58,59,60,67].

### 3.2. Brain Magnetic Studies

#### 3.2.1. Anhysteretic Remanent Magnetization (ARM)

**Figure 1:**
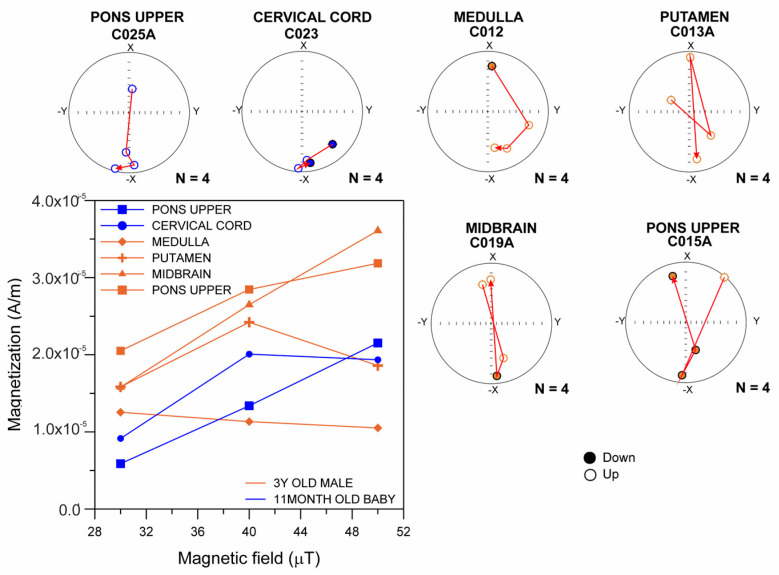
Curves of anhysteretic remanent magnetization (ARM) obtained between 30 and 50 µT, indicating the presence of ferrimagnetic minerals with single-domain particles in MMC an 11-month-old infant and 3-year-old child.

Figure 1 shows the ARM magnetization curves obtained between 30 and 50 µT, indicating the presence of ferrimagnetic minerals with single-domain grain size (0.03–0.1 μm) in an 11-month-old infant and a 3-year-old child. Almost all their samples showed ARM variations at 40 and 50 µT, indicating magnetic NP changes in orientation during the acquisition. Displacements of magnetic particles responding to the combined fields of 100 mT AC and 40 to 50 µT DC are documented in young children.

#### 3.2.2. Brain Samples: SIRM and IRM Measurements

We obtained the SIRM measurements in 164 fresh-frozen brain samples from selected cortical and subcortical regions including sleep and arousal hubs. All selected brain regions exhibited the presence of magnetic particles with SIRM (Table 1), and T1, T2, and T3 magnetic motion behavior was induced by applied magnetic fields of 25–1000 mT AC.

Samples from medullary and mesencephalic reticular formation, nucleus raphe magnus, periaqueductal gray, substantia nigrae, red nucleus, mesopontine tegmental nuclei, and locus coeruleus were included as brainstem samples for the purpose of magnetic studies.

#### 3.2.3. Brain Samples: Saturation Isothermal Remanent Magnetization (SIRM)

**Figure 2:**
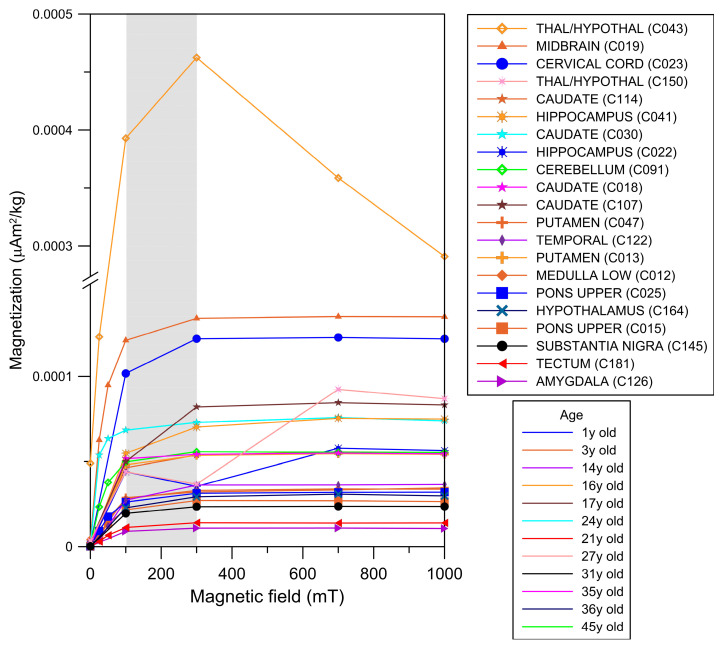
IRM acquisition curves from different brain anatomical areas in males aged 11 months to 45 years. The gray area indicates the mT intensity field at which the ferromagnetic minerals reached IRM saturation values (i.e., magnetization of saturation).

Across the cohort, IRM curves reached saturation upon exposure to magnetic fields of 100 to 300 mT, indicating the presence of low-coercivity magnetic minerals. Brain samples from children showed striking IRM acquisition curve variations between 50 and 300 mT, with decreases in IRM values interpreted as particle movement. This behavior is exemplified in samples from 5 children and 7 young adults in Figure 2.

#### 3.2.4. Brain Samples: Magnetic Components in Equi-Angular Projection Diagrams

**Figure 3:**
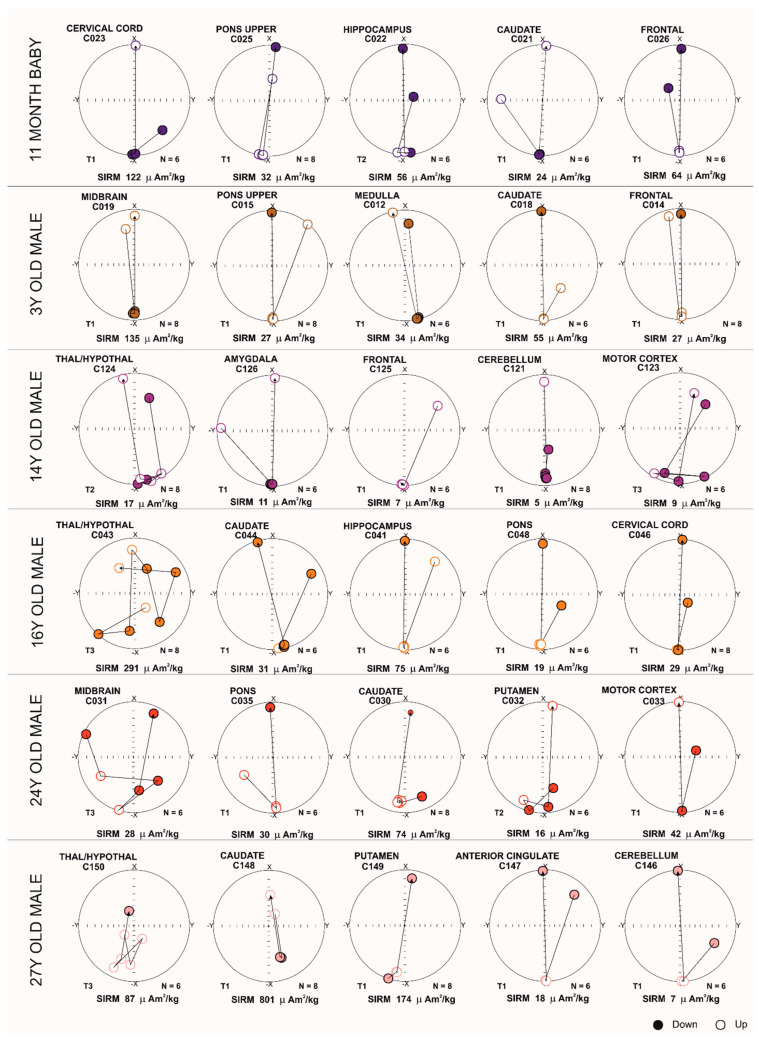
Equi-angular projection diagrams of magnetic vector components of different brain regions in an 11-month-old and subjects of ages 3, 14, 16, 24 and 27 years. T1, T2, and T3 magnetic behavior is correlated with the displacement of magnetic particles.

Magnetic NP orientation changes using equi-angular projection diagrams are displayed in Figure 3. Magnetic field pulses were increased from 25 mT to 1000 mT. Ferrimagnetic materials display a stable magnetic vector direction, while a change in orientation (180°) is expected when an inverse field of 300 mT is applied. Three defined motion behavior groups were identified: T1 displays no changes during magnetization, maintaining an −x orientation of 180 degrees during the IRM acquisition. Characteristically, when a reverse field (300 mT) is applied, T1 inverts its orientation to +x (0 degrees). T2 shows changes in magnetic orientation, rotating ~180 degrees during the magnetization acquisition, with potential changes in the position of the magnetic material near its original position. In sharp contrast, T3 exhibits changes in orientation throughout the acquisition process. Acquisition of magnetization was observed upon exposures to 50 mT fields, and was associated with variations in the magnetic mineral’s position [62].

Figure 3 shows T3 in subcortical teenage and young adult samples, regardless of SIRM. A 14-year-old boy with T2 in thalamus/hypothalamus (SIRM 17 µAm^2^/kg) and T3 in motor cortex (SIRM 9 µAm^2^/kg), was exposed to high concentrations of air and soil metal NPs, and we documented neuropathological hallmarks of AD and PD. The results of this 14-year-old contrast with those of the 16-year-old and 24-year-old with T3 in thalamus/hypothalamus (SIRM 291 µAm^2^/kg) and the T3 in midbrain (SIRM 28 µAm^2^/kg), respectively. The older 27-year-old with T3 in thalamic, hypothalamic regions (SIRM 87 µAm^2^/kg) was a resident in a heavily polluted Mexico state site and simultaneously exhibited AD, PD, and TDP-43 pathology. Interestingly, the 24-year-old with T3 in midbrain and AD and PD neuropathology was an avid motorcycle rider for more than a decade, and his demise related to a motorcycle accident. None of these young subjects carried an APOE4 allele.

### 3.3. Energy-Dispersive X-Ray Spectrometry (EDX) and Brain Transmission Electron Microscopy (TEM)

**Figure 4:**
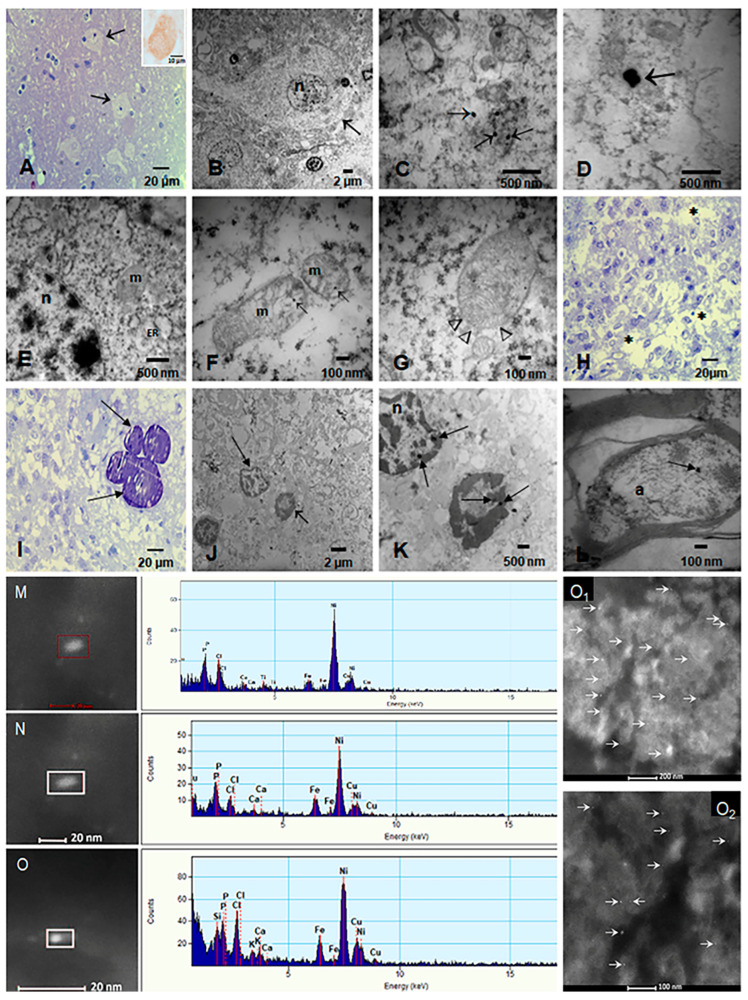
Light and electron microscopy and EDX of targeted sleep and arousal hubs. (**A**) Sixteen-year-old (CO43) toluidine blue 1 μm section of the lateral hypothalamic nucleus (arrows). Insert shows a neuron immunostained with Orexin, brown cytoplasmic product. (**B**) Same subject, EM × 5000, neuron with disrupted cytoplasm and a fragmented cytoplasmic membrane (arrow). (**C**) EM × 50,000 numerous NPs inside fragmented mitochondria and loose in the cytoplasm (arrows) (**D**) Two NPs in the midst of the cytoplasm × 83.300. (**E**) Orexinergic neuron with dilated endoplasmic reticulum (ER) and fragmented mitochondria (m) with matrix NPs. The nucleus also contains NPs (n) × 50,000. (**F**) Mitochondria with fragmented cristae and matrix NPs (arrows) × 133,000. (**G**) Mitochondria with disrupted membranes (arrow heads), containing numerous NPs × 133.000. (**H**) Pineal gland, 21-year-old male, 1 μm toluidine blue shows pinealocyte cords surrounded by loose stroma × 40. (**I**) Pineal with characteristic lamellate calcareous bodies (arrows) × 40. (**J**) EM pineal × 5000, the stroma is fragmented, and two nuclei are identified; the short arrow points to a piknotic unidentified nucleus, the long arrow to a pinealocyte nucleus. (**K**) Same section as J at higher power × 15,000 to show the NPs (arrows)inside both nuclei. (**L**) Axon in subthalamic nucleus with one NP (arrow) × 80,000 (**M**–**O**): Single-domain NPs on the left, corresponding to the EDX on the right: Fe is present in the three NPs. O_1_ and O_2_ show FeNPs in thalamic and lateral hypothalamic nuclei.

EDX from the current study and previous ones in our laboratory [12,13,14,15,16,49] have shown Fe, Ni, Co, Ti, V, Hg, Cu, Zn, Cd, Al, Mg, Ag, Ce, La, Pr, W, Ca, Cl, K, Si, S, Na, and/or Br UFPM/NPs in hypothalamic and thalamic nuclei, pineal gland, hippocampus, caudate, putamen, substantia nigra, tectum, periventricular gray, locus coeruleus, dorsal raphe, raphe magnus, mesencephalic reticular formation, red nucleus, oral pontine reticular nuclei, inferior olivary nucleus, frontal and temporal cortex, and cerebellum (Figure 4). Metals were associated with abrasion and deterioration of automobile catalysts, electronic waste, and rare-earth elements.

Magnetic Fe NPs were localized in neurons of the orexinergic lateral hypothalamic area (orexin immunoreactive, Figure 4A insert), thalamic, noradrenergic (locus coeruleus), dopaminergic nuclei (substantia nigra), cortical, subcortical-including, i.e., caudate, putamen, globus pallidus, amygdala, hippocampus, cuneiform, pedunculotegmental, oral pontine reticular, paramedian raphe and caudal linear raphe nuclei, periventricular gray (PAG), and cerebellum [12,13,14,15,16,49]. UFPM and NPs were identified inside mitochondria, ER, lysosomes, Golgi apparatus and in relationship with heterochromatin and nuclei matrix and pores. In addition, we documented NPs in axons and within myelin sheets and in the pineal gland (pinealocytes, interstitial glial cells, and endothelium) (Figure 4H–K). All subjects in this study exhibited neuropathological hallmarks of AD, PD, and/or TDP-43 associated diseases.

**Figure 5:**
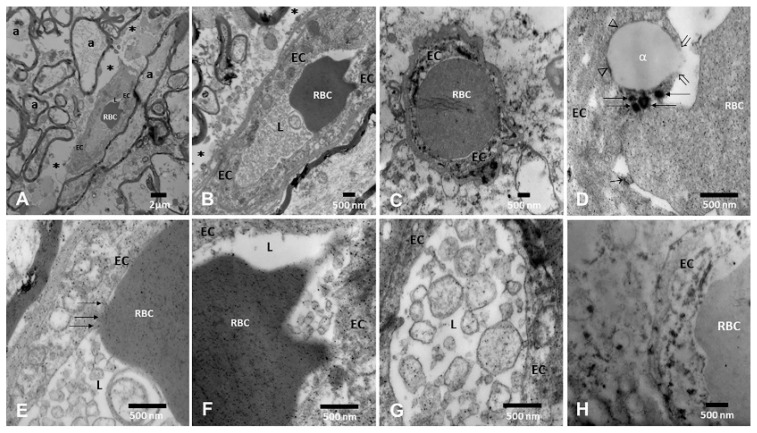
Neurovascular unit pathology in MMC young residents. (**A**). Medullary reticular formation in a 21-year-old × 8330 (C180–C186). A small blood vessel contains one intraluminal red blood cell (RBC). Note the surrounding axons (a) and the loose neuropil spaces (*). (**B**) Same 21-year-old, reticular formation, note the abundant endothelial cell (EC) fragments in the lumen and the extensive perivascular loose neuropils × 15,000. (**C**) Thalamus × 15,000 small vessel with one RBC occupying the entire lumen and in close contact with the EC. (**D**) Periventricular gray PAG × 50,000. Note the close contact between the RBC and the EC lysosomal structures (arrows) and the lipid-like EC accumulation with an opening of the EC membrane integrity (open arrows). (**E**) Dorsal raphe blood vessel × 83,300 with a segment of the RBC closely attached to the EC (arrows) and the presence of luminal EC fragments. (**F**) Medullary reticular formation × 50,000, the RBC shows numerous NPs, and the EC is loaded with NPs. (**G**) A close-up × 50,000 of the luminal membranous structures with abundant NPs. (**H**) Pineal in a 21-year-old, the vessel is filled with NP lysosomes × 50,000.

The neurovascular unit (NVU) pathology associated with the presence of UFPM/NPs is shown in Figure 5. A striking early TEM finding was the transfer of NPs from luminal red blood cells (RBC) to brain capillary endothelium (EC) with ongoing erythrophagocytosis along with endothelial fragments containing UFPM/NPs in the capillary lumen.

## 4. Discussion

Hypothalamic (including orexin immunoreactive neurons), thalamic, and brainstem regions involved in arousal, sleep, narcolepsy/cataplexy, reward processing, addictive and feeding behaviors, and autonomic and motor responses were explored in MMC children and young adults with AD, PD, FTLD, and ALS neuropathology hallmarks. Young urbanites displayed ferrimagnetic, UFPM/NPs with motion behavior in all explored regions.

The early development of fatal neurodegenerative diseases in young megacity urbanites poses an unattended public health challenge and supports the relationship between aberrant proteinopathies, sleep disorders, genetics [11,32] and air pollution [29,30,31,32,33,34,35,36,37,38,39,40,51,52,53,54,55,56,57,58,59,60].

Neurodegenerative diseases and sleep disorders are a global health problem involving alterations in circadian rhythms and psychological stress and are impacted by air pollution [1,2,3,4,5,6]. Insufficient sleep, circadian disruption, artificial light at night, and noise are key factors in self-reported sleep disorders among California teachers [7]. Exposures to outdoor/indoor particulate matter and chemical pollutants, and/or direct exposure to emission sources like tobacco are associated with sleep disorders [68,69,70,71,72,73].

In this study, hypothalamic orexin neurons displayed high concentrations of low-coercivity magnetic minerals, key to NP motion inside critical organelles and cell structures under magnetic fields. Remarkably, among children and young adults, there was a significant subcortical accumulation of magnetic particles, in keeping with the earliest neuropathological location of aberrant proteins [11,12,13,14,15,49] and the development of sleep/awake/autonomic disorders/obesity in young MMC residents [30,31,74,75].

Redox-active, magnetic UFPM/NPs have cytotoxic effects associated with magnetic fluid hyperthermia [76], and with endothelium being vulnerable to the production of reactive oxygen species (ROS), lactate dehydrogenase, and apoptosis [77]. Neurovascular unit damage, endothelial dysfunction, vascular and neural ROS production, magnetic hyperthermia, reduced lysosomal performance, and the intrinsic permeability spectra of ferrimagnetic materials all are strongly dependent on the distribution of local effective magnetic fields and likely synergistically contribute to neural damage in MMC young residents [78,79,80,81,82,83,84].

Three other factors could facilitate the passage of magnetic NPs through neural membranes: the association of NPs with lipopolysaccharides and their electrostatic interactions [85], the impact of saturated lipid membranes in combination with weak magnetic fields [86], and the combination of heavy metals with the primary Fe anthropogenic NPs [87].

It is plausible that the motion under magnetic fields of highly toxic, oxidative, magnetic NPs could result in magnetic hyperthermia, diffusion, convection, residual magnetization, and electromagnetic drift [88,89].

Single-domain iron UFPM/NPs composed of magnetite and maghemite are central to our findings, and their accumulation in the targeted tissues will depend on their hydrodynamic radius and surface charge regulating their time in circulation, accessibility to tissues and cell uptake, and the NPs’ crystallinity and magnetic responses [90].

Moreover, the aggregation of iron oxide NPs and their agglomeration also influence their magnetic and heating properties. It is important to note that interactions of single-domain NPs < 30 nm and protein NPs are potentially relevant to the cytotoxicity of FeNPs in targeted hubs [51,52,91,92,93,94,95]. Magnetic NPs associated with silica were previously seen in MMC samples [94], an intriguing observation in view of the work of Iliasov et al. [92], where Fe NPs coated with Si shells significantly decreased the viability of PC3 cancer cells in a low-frequency alternating magnetic field vs. uncoated NPs. Iliasov et al. [92] discussed that uncoated silica NPs, primarily found in an aggregated form in cells, lose their colloidal stability in an acidic endosomal environment after internalization and become unable to rotate efficiently. Silica shell coating increases NPs’ stability, preventing them from aggregating in endosomes and thus causing significantly more cell damage [92].

We argue that ferrimagnetic particles are a culprit in sleep disorders and their association with aberrant proteinopathies is worrisome in pediatric and young adult populations. The 5G wireless communication technology infrastructure and low orbital satellites could represent a new hazard for millions of people according to Redmayne and Maisch and others [96,97]. Indeed, there is no protection from near-field 5G for the public [98], and thus, worldwide populations accumulating magnetic particles in their brains could be at high risk.

Neurodegenerative diseases and sleep disorders are increasing around the world [7,8,9,10,33,34,35,36,37,38,99,100,101,102,103,104,105,106,107,108,109], and the relationship between brain Fe accumulation and high risk, i.e., α-synucleopathies, REM sleep disorders are seen on brain 3T MRI [45,99]. The literature associating cortical and subcortical neuronal populations, neurodegeneration, REM behavior disorders, chronic insomnia, and obesity with air pollution and electromagnetic radiation is relevant to neurologists, psychiatrists, sleep experts, internists, pediatricians, and psychologists [20,21,22,23,51,52,53,110,111,112,113,114,115].

The work of Oh et al. [110] is pertinent to megacity residents where the subcortical pathology is associated with sleep disturbances in the early stages of neurodegenerative diseases, essential for what we are examining in MMC pediatric and teen populations. Strikingly, the autonomic system (ANS) and the circadian rhythm are also involved [116,117,118,119,120].

The complexity and interaction of the CNS/ANS contributes to the impact of magnetic NPs, and urban children could be significantly affected. Autonomic dysfunction and sleep alterations should be identified in the medical history of pediatric populations living in polluted cities.

Further, the inhalation of diesel exhaust particles [121] increases weight in exposed mice, is associated with increased expression of hypothalamic gamma-aminobutyric acid (GABA) type B receptor and orexin receptors, and a repressed HPA axis, an important observation in view of the association between sleep, depression, cognition deficits, and obesity in the USA [122]. Of deep concern is pediatric obesity and metabolic derangement, a problem studied in MMC children by our group [74,75].

The work of Koshko et al. [123] is also relevant to urban children, demonstrating that prenatal environmental contaminants directly impact the hypothalamus with impaired metabolic programming. Compounding the problems for MMC children, we have demonstrated the presence of magnetic anthropogenic UFPM/NPs in fetal brains and in placentas of all gestational ages [16].

The early presence of magnetic particles in critical brain regions, such as the diencephalon, certainly could contribute to the early-life disruption of metabolic programming described by Koshko et al. [123]. Tamayo-Ortiz et al. [111] have shown that a 10 µg/m^3^ increase in PM_2.5_ in MMC adolescents was associated with an obesity OR of 3.53 (95% CI: 1.45, 8.58) and 3.79 (95% CI: 1.40, 10.24) for 2006 and 2012, respectively.

Three major limitations apply to our current findings: i. Autopsy forensic studies are focused on MMC residents exposed for decades to complex mixtures of air pollutants, plus potential neurotoxicants from water, soil, foods, and industrial and indoor pollution. ii. Our cohort was almost entirely male. iii. Although we had access to the entire autopsy, we lacked clinical histories and occupational records. On the other hand, we have been studying *clinically healthy* children and young adults for the last two decades and written extensively on their inflammatory status, plasma and CSF neurodegenerative biomarkers, progressive cognition deficits, and significant brain atrophy in the absence of well-known risk factors, including tobacco, alcohol consumption, and occupational exposures [24,25,26,27,28,29,30,31,32,74,75].

## 5. Conclusions

Our data suggest that the early development of AD, PD, and TDP-43 pathology is an early link between neurodegeneration and sleep disorders.

We foresee the importance of clinical, laboratory, and brain MRI studies focused on sleep, awake, autonomic, and reward hubs to bridge the gap between anthropogenic air pollution, neurodegeneration, sleep, and autonomic and eating disorders in highly exposed children and young adults.

We need to identify children at higher risk and monitor environmental UFPM and NPs emissions and exposures to magnetic fields. Early pediatric screening for sleep disorders, cognition, neuropsychiatric status, autonomic function, and BMI is important in predicting outcomes and risk.

Individual radio-frequency electromagnetic field (RF-EMF) exposures have increased in the last few years, and the harmful health effects are raising serious concerns about new technologies and exposures from intrauterine life and their short- and long-term brain health consequences. As health providers, we need to understand the biological effects of RF-EMF and to listen to EMF hypersensitive individuals with sleep complaints associated with mobile telecommunication—a situation that needs to be explored in children and adolescents using cell phones.

Pediatricians, toxicologists, neuropathologists, and health workers across the world need to protect their populations. Monitoring systems for NPs in air, foods, soil, plants, urban dust, and occupational settings, in addition to exposures to RF-EMF, are imperative.

Magnetic UFPM/NPs are an environmental early-life threat.

## Figures and Tables

**Table 1:** One hundred and sixty-four brain regions with saturation isothermal remanent magnetization (SIRM) measurements. Average age of the individuals in this study was 35.4 ± 21.9 years, including 9 children, aged 12.8 ± 6.4 years.

	n	Average Age	Weigh	ARM_50_	SIRM	IRM-_300_	S_300_
				μAm^2^/kg	μAm^2^/kg	μAm^2^/kg	AD *
All samples	164	31.1 ± 20.5	0.7 ± 0.3	0.59 ± 2.80	30.7 ± 63.4	32.9 ± 89.0	1.0 ± 0.5
All adult samples	109	40.9 ± 18.3	0.7 ± 0.3	0.68 ± 3.50	30.7 ± 88.6	31.9 ± 97.8	1.0 ± 0.5
All children samples	55	12.0 ± 6.5	0.7 ± 0.4	0.41 ± 0.70	30.6 ± 44.4	35.0 ± 71.2	1.0 ± 0.1
Cortical adults	47	43.9 ± 18.5	0.9 ± 0.3	0.18 ± 0.14	15.0 ± 13.7	14.6 ± 13.6	1.0 ± 0.1
Cortical children	18	12.8 ± 6.2	0.8 ± 0.5	0.24 ± 0.26	18.7 ± 15.7	21.7 ± 24.0	1.0 ± 0.1
Subcortical adults	60	38.8 ± 18.0	0.6 ± 0.3	1.08 ± 4.70	43.5 ± 117.0	43.5 ± 117.0	1.0 ± 0.1
Subcortical children	35	11.7 ± 6.7	0.6 ± 0.3	0.51 ± 0.85	37.3 ± 53.0	42.4 ± 86.6	1.0 ± 0.1
T1 Motion adults	119	45.2 ± 20.7	NA	0.64 ± 3.30	29.0 ± 75.0	28.5 ± 74.0	1.0 ± 0.1
T1 Motion children	47	12.0 ± 6.6	NA	0.33 ± 0.37	26.0 ± 28.5	25.5 ± 28.0	1.0 ± 0.1
T2 Motion adults	6	50.8 ± 27.3	NA	1.35 ± 2.80	96.5 ± 184.0	127.0 ± 262.0	0.9 ± 0.1
T2 Motion children	4	8.5 ± 7.7	NA	0.37 ± 0.19	30.0 ± 18.3	23.4 ± 8.5	0.9 ± 0.1
T3 Motion adults	3	25.0 ± 1.7	NA	0.35 ± 0.14	87.0 ± 113.0	40.4 ± 30.0	0.9 ± 0.1
T3 Motion children	5	15.2 ± 1.09	NA	1.22 ± 1.90	86.8 ± 113.0	133.3 ± 217	0.9 ± 0.1
Frontal adults	14	42.0 ± 19.6	0.9 ± 0.3	0.15 ± 0.10	13.8 ± 13.0	13.5 ± 12.7	1.0 ± 0.1
Frontal children	9	12.6 ± 6.3	1.0 ± 0.4	0.26 ± 0.33	16.8 ± 19.0	15.8 ± 18.3	0.9 ± 0.1
Temporal adults	21	42.6 ± 18.2	0.9 ± 0.3	0.23 ± 0.16	17.5 ± 16.6	17.0 ± 16.4	1.0 ± 0.1
Temporal children	7	11.6 ± 6.7	0.7 ± 0.3	0.18 ± 0.08	20.2 ± 10.6	29.7 ± 31.8	1.0 ± 0.1
Cingulate anterior adults	11	42.5 ± 18.5	0.8 ± 0.2	0.13 ± 0.06	10.5 ± 4.2	9.9 ± 4.3	0.9 ± 0.1
Cingulate ant children	2	17	0.8 ± 0.2	0.15 ± 0.10	10.5 ± 6.0	10.2 ± 5.7	1.0 ± 0.1
Hippocampus adults	3	59.6 ± 30.0	1.0 ± 0.3	0.12 ± 0.03	9.7 ± 3.8	8.3 ± 1.9	0.9 ± 0.1
Hippocampus children	4	11.7 ± 7.3	0.4 ± 0.2	0.40 ± 0.25	40.9 ± 30.0	35.2 ± 27.3	0.9 ± 0.1
Thalamus/Hypothalamus adults	5	29.0 ± 6.2	0.6 ± 0.4	0.34 ± 0.27	29.4 ± 32.2	26.8 ± 26.7	1.0 ± 0.1
Thalamus/Hypothalamus children	3	15.6 ± 1.5	0.7 ± 0.3	1.8 ± 2.76	105.0 ± 161.0	180 ± 291	1.0 ± 0.1
Motor cortex adults	13	38.2 ± 15.0	0.8 ± 0.3	0.18 ± 0.11	15.5 ± 14.2	15.2 ± 13.8	1.0 ± 0.1
Motor cortex children	5	13.2 ± 6.9	0.7 ± 0.4	0.51 ± 0.60	35.3 ± 49.4	33.7 ± 48.3	0.9 ± 0.1
Brainstem sleep hubs adults	12	37.0 ± 21.0	0.5 ± 0.2	0.2 ± 0.09	16.5 ± 8.2	16.5 ± 8.9	1
Brainstem sleep hubs children	5	5.2 ± 6.0	0.4 ± 0.3	0.65 ± 0.60	49.3 ± 48.0	49.0 ± 48.0	1
Caudate adults	12	36.5 ± 15.0	0.6 ± 0.1	3.5 ± 10.30	93.5 ± 223.0	92.0 ± 220.0	1.0 ± 0.1
Caudate children	5	11.0 ± 8.2	0.6 ± 0.2	0.97 ± 0.71	74.7 ± 57.0	73.4 ± 56.0	1
Putamen adults	12	40.8 ± 17.2	0.7 ± 0.2	0.43 ± 0.95	26.0 ± 47.4	25.4 ± 47.2	1.0 ± 0.1
Putamen children	6	14.8 ± 5.8	0.6 ± 0.2	0.25 ± 0.23	21.3 ± 19.3	21.1 ± 19	1.0 ± 0.1
Cerebellum adults	13	37.6 ± 17.4	0.8 ± 0.3	0.23 ± 0.16	20.2 ± 24.6	19.9 ± 24	1.0 ± 0.1
Cerebellum children	9	12.8 ± 6.4	0.8 ± 0.3	0.17 ± 0.08	12.9 ± 6.2	12.5 ± 6.2	1.0 ± 0.1

AD * dimensionless.

## Data Availability

All data are included in the manuscript.

## Supplementary Materials

The following supporting information can be downloaded at: https://www.mdpi.com/article/10.3390/toxics13040284/s1, Table S1: Forensic autopsies with APOE and neuropathological diagnosis. Cases were examined with H&E, PHF-tau8 phosphorylated at Ser199-202-Thr205, α-synuclein phosphorylated at Ser-129, LB509 and TDP-43 mab2G10, and rabbit polyclonal Ab recognizing N-terminal TDP-43.
