# Supplementary material for: Sleep and Arousal Hubs and Ferromagnetic Ultrafine Particulate Matter and Nanoparticle Motion Under Electromagnetic Fields: Neurodegeneration, Sleep Disorders, Orexinergic Neurons, and Air Pollution in Young Urbanites
Source: Toxics. 2025 Apr 8;13(4):284. doi: 10.3390/toxics13040284 (PMC12030987; doi:10.3390/toxics13040284)
Supplement: Supplementary file 1 [file toxics-13-00284-s001.zip › toxics-3503514-supplementary.pdf]

Supplemental Table S1: Forensic autopsies with APOE and neuropathological diagnosis. Cases were examined with H&E, PHF-tau8 phosphorylated at Ser199-202-Thr205, α-synuclein phosphorylated at Ser-129, LB509 and TDP-43 mab2G10 and rabbit polyclonal Ab recognizing N-terminal TDP-43.

ID#	AGE	SEX	APOE	pTau stage	Abeta Phase	pTau NTs	pTauNFT	OBαS	LC pTau	SN pTau	SNαS	TDP-43 BRAINSTEM
C001	25	1	0	2	2	2	2	2	0	0	1	0
C 002	55	1	0	3	2	1	1	0	0	0	0	0
C003 C004	55	1	0	5	4	2	2	1	0	0	0	0
C005	90	0	0	4	3	1	1	1	1	1	1	2
C006	41	1	0	5	3	1	1	1	0	1	1	1
C007	29	0	0	2	2	1	1	1	0	1	1	1
C008	39	1	0	3	2	2	1	1	0	0	0	0
C009	53	1	0	4	3	1	2	1	1	1	1	1
C010	27	1	0	2	2	1	1	1	0	2	1	1
CO11	65	1	0	4	3	1	1	1	1	1	1	0
CO12-CO19	3	1	0	2	0	1	1	1	0	1	0	0
CO20-CO27	1	1	0	1	2	1	0	0	0	1	0	1
CO28-CO35	24	1	0	2	2	1	1	1	0	0	0	0
CO36-CO40	13	0	0	2	2	1	0	1	0	1	1	0
CO41-CO48	16	1	0	2	2	1	1	1	0	1	0	1
CO49-C056	25	0	0	2	2	1	0	1	0	1	0	0
C085-C087	17	1	0	1	2	0	0	0	0	0	0	0
C088-C093	45	1	0	4	3	1	1	1	1	1	0	0
C094-C099	54	1	0	3	2	1	0	0	1	1	0	0
C0100-104	17	1	0	3	2	1	1	1	1	1	0	1
C105-C112	35	1	0	2	2	1	1	1	1	1	0	0
C113-C120	17	1	0	2	2	0	0	0	0	1	0	0
C121-C126	14	1	0	2	2	1	1	1	1	1	0	0
C127-134	58	1	0	3	2	1	0	0	1	1	0	0
C135-C138	22	0	0	2	2	1	1	1	1	1	1	1

C140-C145	31	1	0	2	2	1	1	0	0	0	0	0
C146-C150	27	1	0	2	2	1	1	1	0	0	0	0
C151-CC155	20	1	1	2	2	2	2	1	0	1	1	0
C156-C160	63	0	0	4	3	2	1	0	0	1	1	0
C161-C171	36	1	1	5	3	0	0	0	2	3	1	1
C172-C174	72	1	0	4	3	1	1	1	1	1	1	1
C175-C179	18	1	0	2	2	1	1	0	1	1	1	0
C180-C186	21	1	0	3	2	1	1	1	1	0	1	1
C187-C194	77	0	0	4	3	2	1	2	2	1	1	2

Gender: 0=female, 1=male. APOE 0=3/3, 1=3/4

AD staging pτ Stage: 0=absent, 1= pre-tangle stages a-c, 2= pre-tangle stages 1a,1b, 3=NFT stages I, II, 4=NFT stages III-IV, 5=NFT stages V-VI

AD staging Aβ Phase: 0=absent, 1=basal temporal neocortex, 2=all cerebral cortex, 3=subcortical portions forebrain, 4=mesencephalic components, 5=Reticular formation and cerebellum.

Substantia nigrae pτ was evaluated as none=0, pre-tangles, positive neurites, and tangles using the PHF-tau8 phosphorylated at Ser199-202-Thr205 =1(Innogenetics, Belgium, AT-8 1:1000).

Substantia nigrae α-S was evaluated as none=0 and neuronal immunoreactive (IR) aggregates in the somato-dendritic compartment, cytoplasmic inclusions, core-halo Lewy bodies and dystrophic neurites (Lewy neurites)=1, using α-synuclein phosphorylated at Ser-129, LB509 (In Vitrogen, Carlsbad, CA 1:1000)

* Brainstem TDP-43 using two Ab was evaluated as none=0 and dash-like IR particles in the vicinity of the cell nucleus, with or without complete loss of nuclear TDP-43 expression and somatic skein-inclusions=1, using mab2G10 (Roboscreen GmbH, Leipzig, Germany 1:1000) and Proteintech TDP-43 rabbit polyclonal antibody recognizing the N-terminal TDP-43 (Proteintech #10782-2-AP).
